# Encapsulation of Fresh *Spirulina* Biomass in Alginate Spheres for Yogurt Fortification

**DOI:** 10.3390/microorganisms13071641

**Published:** 2025-07-11

**Authors:** Domenico Siclari, Maria Rosaria Panuccio, Rossana Sidari

**Affiliations:** Department of Agraria, *Mediterranea* University of Reggio Calabria, 89124 Reggio Calabria, Italy; siclarid1999@libero.it (D.S.); mpanuccio@unirc.it (M.R.P.)

**Keywords:** *Spirulina*, spherification, lactic acid bacteria, yogurt, functional food, viable *Spirulina* formulation

## Abstract

A new spherification of *Spirulina* (*Arthrospira platensis*) was developed for its use as a food supplement. The novelty of this study is the incorporation of fresh *Spirulina* biomass into alginate spheres formulated with 3% sodium alginate and 1.5% calcium lactate and its addition into yogurt. The spheres and the fortified yogurt were stored at 4 °C for 15 days. The viability of *Spirulina*, either in contact with the yogurt or not, was evaluated both by OD_550nm_ measurements and microscopic observations. Furthermore, the effect of *Spirulina* spheres on *Streptococcus thermophilus* and *Lactobacillus bulgaricus* was evaluated by enumerating them in standard media. *Spirulina* retained its viability for up to 15 days when stored separately from the yogurt matrix. *Spirulina* had a stimulating effect on the lactic acid bacteria: after 15 days, *L. bulgaricus* and *S. thermophilus* showed a load increase of 2.66% and 1.64%, respectively, compared to the load detected in the unfortified yogurt. Our study has demonstrated the technical feasibility of producing fresh *Spirulina* spheres, which can be used alone or added to food preparation. Nevertheless, additional investigations—including quantitative assessment of bioactive compounds and comprehensive sensory analysis—are essential to validate the methodology and support its scalability.

## 1. Introduction

The cyanobacterium *Arthrospira platensis*, *Spirulina*, is defined as a superfood thanks to its high content in proteins, carbohydrates, fats including polyunsaturated ω3 and ω6 fatty acids, vitamins, pigments, and phenolic compounds. Moreover, *Spirulina* has beneficial health effects by protecting against oxidation and aging, cardiovascular diseases, hypertension, renal failure, cancer, and male infertility [1,2].

Most of the *Spirulina* biomass produced today is sold as a food supplement in the form of dried powder, flakes or capsules. Consumers are becoming increasingly health-conscious and are seeking functional products that offer nutritional benefits beyond basic sustenance. Therefore, the production of functional foods/beverages with the addition of this microalga such as yogurt and cheese, baked goods, pasta, snacks, and ice cream is increasing, as is the use of *Spirulina* in cooking as an ingredient for various sweet and savory preparations [3].

This study introduces a simple and cost-effective spherification method designed to preserve the viability of fresh *Spirulina* for potential use in functional foods, particularly yogurt. Therefore, the external gelation, or direct spherification method, was selected in preference to other encapsulation techniques, which may potentially preserve the properties of the fresh *Spirulina* unaltered. This selection avoided extreme factors, such as high temperature, which would impair the composition or viability of the fresh material. Furthermore, this choice was justified by the simplicity, speed, and low cost of the technique.

A very recent study reports on the fortification of wheat noodles with fresh *Spirulina* microcapsules [4]. To the best of our knowledge, to date no studies have combined the encapsulation of viable *Spirulina* biomass with the fortification of yogurt.

In this work, we have used live *Spirulina*; therefore, it fills the gap existing in the literature that broadly reports the fortification of dairy products with dead *Spirulina* biomass usually added freely to the products. As regards *Spirulina*-fortified novel dairy products that contain both the bioactive microalga compounds and beneficial lactic acid bacteria (LAB), *Spirulina* is generally used as a dehydrated, freeze-dried and spray-dried biomass [5,6,7].

The high moisture content of the fresh biomass limits its use in the food industry [8]; therefore, biomass dehydration allows the shelf life of *Spirulina* to be significantly extended and allows for its easy incorporation into foods and drinks. However, regardless of the drying method used and how well it is managed, there is a loss of various thermolabile and/or easily oxidizable nutritional and bioactive compounds when compared to the fresh product [9,10]. For this reason, many companies in recent years have begun to market fresh biomass, thus trying to offer consumers a product with unchanged nutritional and functional characteristics [11]. Generally, these methods are complex and expensive; moreover, the consumer may not accept the *Spirulina* food/beverage produced by the above methods due to the overly intense blue-green color and the fishy taste and smell conferred by the addition of *Spirulina* [12,13,14,15]. The embedment of the *Spirulina* biomass using various coating matrices could be one effective solution to these problems. For this purpose, different encapsulation techniques such as liposomes and extrusion (or spherification) have been applied to *Spirulina* powder [16,17,18,19,20].

The aims of this research were the production of spheres of fresh, living *Spirulina* to be used on their own as a food supplement, the fortification of an experimental yogurt in order to verify the effect of the *Spirulina* spheres on the LAB, and the verification of the continued viability of *Spirulina* in the spheres either in contact with the yogurt or not.

## 2. Materials and Methods

### 2.1. Arthrospira platensis and Growth Conditions

The strain of *Arthrospira platensis* ULC 445 was purchased from BCCM/UCL Cyanobacteria Culture Collection of the University of Liège (Liège, Belgium).

To obtain broth cultures of *Spirulina*, the strain was inoculated at 1% in the Zarrouk medium [21] (pH 9.0) and incubated in a growth chamber (M120-RHL, MPM Instruments s.r.l., Bernareggio, Italy) at 27 °C illuminated by red and blue LEDs with 100 µmolphoton·m^−2^·s^−1^ photon flux under a photoperiod light/dark of 12:12 h and thrice daily shaking for 13 days.

### 2.2. Spirulina Spheres Production

**Figure 1:**
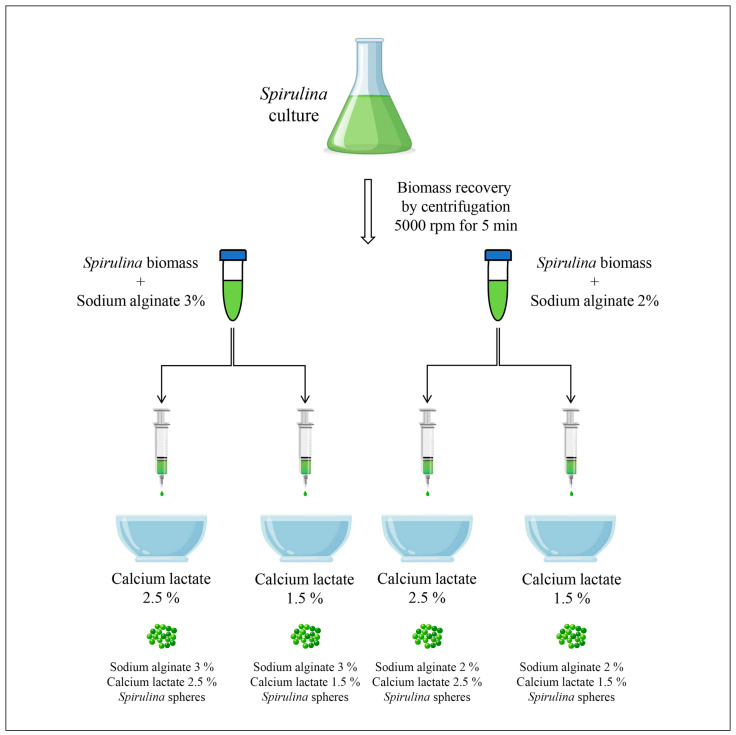
Graphical representation of the experimental design for different *Spirulina* spheres formulations: sodium alginate 3% and calcium lactate 2.5%, sodium alginate 3% and calcium lactate 1.5%, sodium alginate 2% and calcium lactate 2.5%, and sodium alginate 2% and calcium lactate 1.5%.

To produce *Spirulina* spheres, sodium alginate and calcium lactate (Special Ingredients, Savona, Italy) were used. In order to establish the best concentrations of sodium alginate and calcium lactate, a preliminary direct spherification trial was carried out (Figure 1).

For this purpose, sterile sodium alginate solutions at 3% and 2%, calcium lactate solutions at 2.5% and 1.5%, and *Spirulina* broth cultures grown under the conditions reported in Section 2.1 were used. Four combinations of sodium alginate and calcium lactate were considered: 3% sodium alginate either with 2.5% or 1.5% calcium lactate and 2% sodium alginate either with 2.5% or 1.5% calcium lactate. The spherification process was carried out under a laminar flow hood using materials and tools properly sterilized in an autoclave or sanitized with denatured ethyl alcohol (70% *v*/*v*).

The *Spirulina* broth culture was centrifuged for 15 min at 5000 rpm and 4 °C. Then, the supernatant was removed and the sodium alginate solution—3% or 2%—was added and mixed into the *Spirulina* biomass using a sterile steel spatula until homogenous mixtures were obtained. Subsequently, each mixture was dripped from a height of 8 cm using a 10 mL sterile syringe without a needle (diameter 2 mm) into two calcium lactate baths—2.5% or 1.5%—and stirred at a speed of 200 rpm.

The spheres formed instantly when in contact with the calcium lactate bath and they visually resembled roe. They remained in the calcium lactate bath for approximately 30 s, after which they were removed and rinsed with sterile deionized water to remove the calcium lactate residue.

Fifty grams of spheres for each combination of sodium alginate and calcium lactate concentration were obtained. Twenty spheres for each type of sodium alginate and calcium lactate combination were taken randomly and analyzed by ImageJ (https://imagej.net/ij/docs/) to measure their diameter. Briefly, the images of the samples together with a ruler were captured by a Nikon D700 camera (Nikon, Corporations, Tokyo, Japan); then, they were calibrated before measuring the diameters using ImageJ tools. Moreover, they were qualitatively evaluated by macroscopic observation during stirring and by manual compression.

### 2.3. Yogurt Preparation

To prepare the yogurt, semi-skimmed UHT milk (Arborea, Oristano, Italy) and a plain yogurt (Spesotti, Coop, Casalecchio di Reno, BO, Italy) were used. The LAB composition of the plain yogurt reported on the label was *Lactobacillus bulgaricus* and *Streptococcus thermophilus*.

Briefly, 1 L of milk was mixed with approximately 125 mL of the commercial yogurt. Then, the mixture was divided into 125 mL glass jars that were closed with screw lids, incubated at 42 °C for 8 h, stored at 4 °C for 12 h. Then, part of yogurt was fortified with *Spirulina* spheres and part of it remained unfortified and used as a control. For each analysis time and each type of sample (fortified and unfortified) three replicates were prepared.

### 2.4. Fortification of Yogurt with Spirulina Spheres (Main Trials)

**Figure 2:**
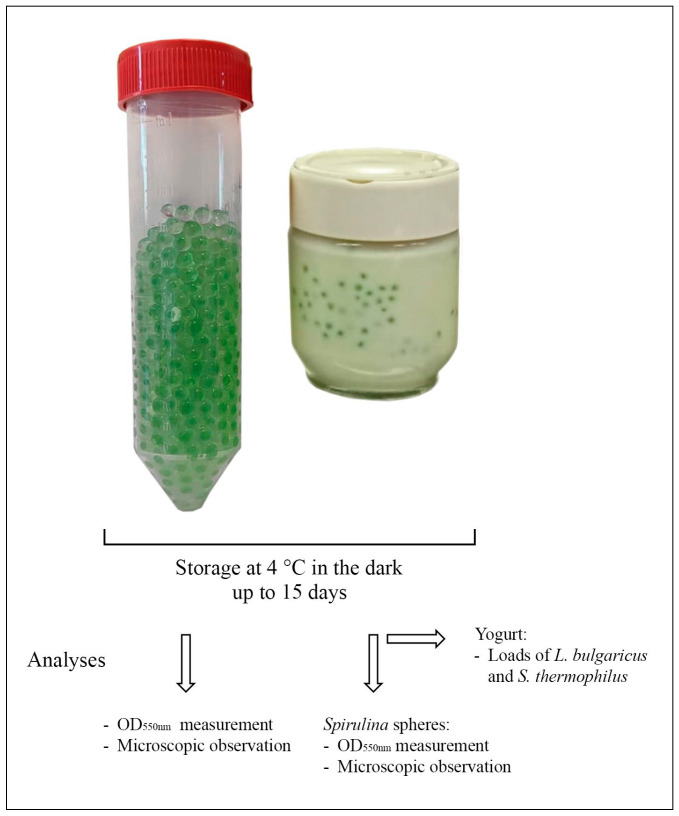
*Spirulina* spheres and yogurt fortified with *Spirulina* spheres, storage conditions, and analyses carried out up to 15 days.

Based on the results of the preliminary trial, the concentration of 3–1.5% of sodium alginate and calcium lactate, respectively, was chosen and used to carry out the main trial. The spheres obtained (250 g) were partly stored in sterile Falcon tubes (control samples) and partly added to yogurt (Figure 2).

Table 1 reports the sample types used and their acronyms. For each yogurt jar, 10 g of *Spirulina* spheres containing 0.76% *Spirulina* biomass were added (samples labeled Y+S).

Yogurt without spheres (samples labeled Y) and *Spirulina* spheres not in contact with yogurt (samples labeled S) were used as controls. All samples were prepared in triplicate. All samples were stored in the dark at 4 °C and analyzed by destructive method (different samples for each time point) across 15 days.

### 2.5. pH and LABs Enumeration

After mixing with a sterile spatula to homogenize the sample, the pH of the yogurt Y and Y+S was measured in triplicate using a pH meter with a probe for semi-solid foods (Hanna Instruments HI 99161, Villafranca Padovana, PD, Italy) at 0, 2, 6, 8, 10 and 15 days.

The yogurt samples—Y and Y+S—were homogenized (1:10) in 0.9% NaCl (*w*/*v*) solution using a Stomacher type homogenizer (Astori Tecnica, Poncarale, BS, Italy) for 2 min at maximum speed, serial diluted and subsequently plated by spreading technique onto De Man–Rogosa–Sharpe (MRS) agar using MRS broth (BioMaxima, Lublin, Poland) and bacteriological agar (18 g L^−1^—VWR International, Leuven, Belgium) for *L. bulgaricus*, and M17 agar (Liofilchem, Roseto degli Abruzzi, TE, Italy) for *S. thermophilus*. In particular, the MRS medium was acidified with lactic acid up to a value of 5.4 [22]. To analyze the Y+S yogurt samples, the *Spirulina* spheres were removed using a sterile slotted spoon and the yogurt was analyzed as previously described. All plates were incubated in anaerobic jars and sachets (Thermo Scientific^TM^ rectangular jar and AnaeroGen^TM^ compact sachet, Thermo Fisher Scientific, Waltham, MA, USA) at 37 °C for 48–72 h. At the end of the incubation, the colonies were counted and analyzed to obtain Log CFU/g. The analyses were performed in triplicate at 0, 2, 6, 8, 10 and 15 days.

The recovered *Spirulina* spheres from the Y+S samples were used to verify the viability of the *Spirulina* (see Section 2.6).

### 2.6. Spirulina Viability Check

The *Spirulina*’s viability was assessed on both S samples (spheres stored in the dark at 4 °C) and Y+S samples (spheres recovered from yogurt stored in the dark at 4 °C) using Zarrouk medium as control. It was measured spectrophotometrically in triplicate by recording the absorbance at 550 nm (OD_550nm_) using the Spectrophotometer UV-1800 (Shimatzu, Kyoto, Japan) (samples and control volume of 2 mL, plastic cuvette with path length of 10 mm) at T0 (immediately after the preparation of spheres and their addition to the yogurt) and at two days after each analysis time (2, 6, 8, 10, and 15 days), therefore at 2, 4, 8, 10, 12, and 17 days of incubation at the condition reported in Section 2.1. For Y+S samples, the spheres were separated from the yogurt by a sterile slotted spoon, rinsed several times with sterile deionized water, and then subjected to the following procedure.

At each analysis time, both the S and Y+S *Spirulina* spheres were homogenized (1:10) in 0.9% NaCl (*w*/*v*) solution by a Stomacher type homogenizer (Astori Tecnica, Poncarale, BS, Italy) for 2 min at maximum speed. Subsequently, the homogenates were inoculated at 5% in Zarrouk medium and incubated in a growth chamber under the same conditions described for *Spirulina* cultivation. Moreover, *Spirulina*’s viability was verified by monitoring its growth with daily macroscopic observation, and with microscopic observation without staining procedures with 20× magnification by using an optical microscope (Olympus BX53, Tokyo, Japan).

### 2.7. Statistical Analysis

The analyses were carried out by SPSS program (version 15.0) considering *p* < 0.05. One-way ANOVA was performed for sphere diameter data; two-way ANOVA test was performed on the entire dataset to analyze the interactions between pH, time, and Y and S treatments, between LAB load, time, and Y and S treatments, and for Y and S *Spirulina* culture spectrophotometer data.

## 3. Results

### 3.1. Preliminary Trial

Four types of spheres were obtained that differed from each other in the concentrations of sodium alginate (3–2%) and calcium lactate (2.5–1.5%). Table 2 shows the diameter and the qualitative characteristics of the spheres obtained with the two concentrations of sodium alginate and the two of calcium lactate.

The highest sodium alginate and calcium lactate combination significantly increased (*p* < 0.05) the diameter of the spheres compared to the other combinations. The combinations 3–1.5% and 2–1.5% had smaller diameters than the 3% and 2.5% combination and did not differ significantly from each other. The spheres with 2–2.5% combination were significantly different (*p* < 0.05) from spheres with 3–1.5% and 2–1.5% combination of sodium alginate and calcium lactate. Concerning the qualitative characteristics of the spheres, the alginate 3% and calcium lactate 2.5% led to the formation of spheres that remained undamaged during the stirring in the calcium bath and that were hard and completely solidified. The spheres obtained using the combination 3–1.5% and 2–2.5% of sodium alginate and calcium lactate gave spheres which remained undamaged during the stirring in the calcium bath, fairly resistant to manual compression, but which were not completely solidified, and released liquid on being broken. Therefore, they were characterized by a thin external layer and a liquid core. The combination 2–1.5% of sodium alginate and calcium lactate formed spheres easily breakable both during the stirring in the calcium bath and by manual compression.

The spheres obtained from 3–2.5% of sodium alginate and calcium lactate, respectively, were discarded for their size and their behavior when crushed, while the spheres obtained from 2–1.5% of sodium alginate and calcium lactate, respectively, were discarded for their fragility. Although both the spheres from combination 3–1.5% and 2–2.5% of sodium alginate and calcium lactate exhibited the best size and qualitative characteristics (Table 2), those with 3–1.5% of sodium alginate and calcium lactate, respectively, were chosen because the lower concentration of calcium lactate would theoretically make the taste of the spheres less bitter.

### 3.2. Main Trials—pH and LABs

Table 3 reports the pH values of the yogurt and the yogurt fortified with *Spirulina* spheres over the 15-day period. For both the Y and Y+S samples, a significant reduction (*p* < 0.05) was observed between day 0 and the subsequent analysis times. The addition of *Spirulina* and the progress of time significantly affect (*p* < 0.05) the pH of the yogurts. The addition of *Spirulina* spheres to yogurt determined significant pH differences at 2 days (*p* < 0.05) compared to the unfortified yogurts at 6, 8, 10, and 15 days. Moreover, the progress of time significantly affected (*p* < 0.05) the pH of fortified samples at 2, 6, and 15 days.

**Figure 3:**
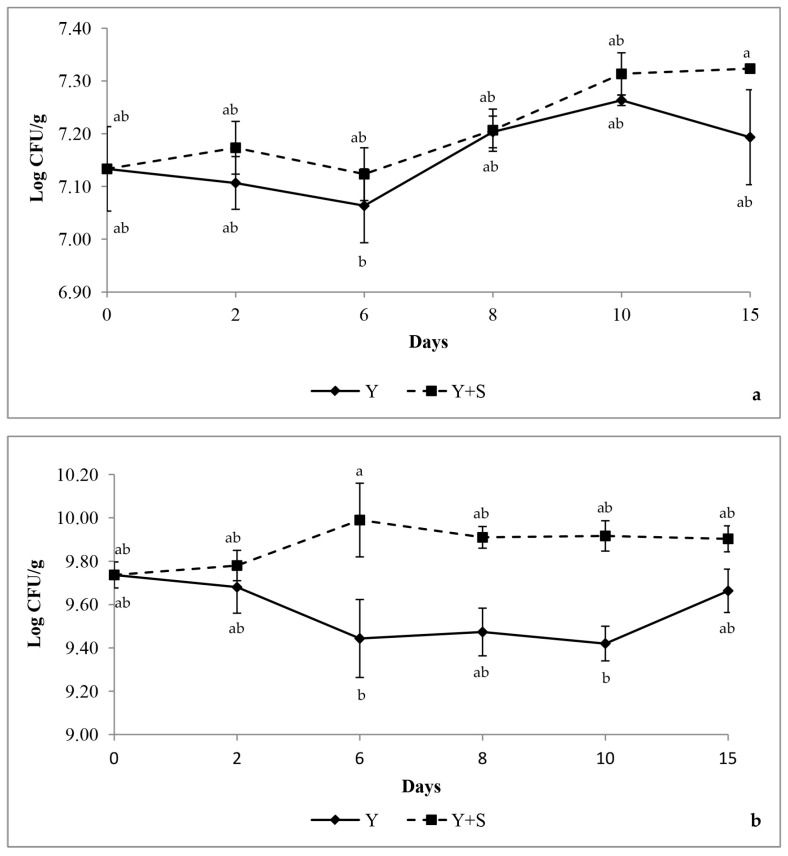
Increase in load of *L. bulgaricus* grown in MRS agar (**a**) and of *S. thermophilus* in M17 agar (**b**) detected in fortified yogurt fortified with *Spirulina* spheres (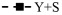) compared to the control yogurt samples (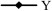) across 15 days. Values are mean ± standard deviation. Means with different superscript letters are significantly different (*p* < 0.05).

Figure 3 shows the loads of *L. bulgaricus* (a) and *S. thermophilus* (b) of Y and Y+S at the different analysis times. The incorporation of *Spirulina* promoted the growth of LABs, as demonstrated by the higher colony counts observed in the fortified samples relative to the controls. Indeed, the LAB loads of Y+S were higher than those of Y, although the differences were not always significant. At the end of the storage time, *L. bulgaricus* showed a significant (*p* < 0.05) 2.66% load increase in Y+S compared to a 0.84% increase in Y, while *S. thermophilus* showed a 1.64% load increase in Y+S compared to a 0.82% decrease in Y and significant differences (*p* < 0.05) at 6 days.

### 3.3. Main Trials—Viability of Spirulina Embedded in Alginate Spheres

Table 4 shows the optical densities against time of the *Spirulina* embedded in S and Y+S spheres.

**Figure 4:**
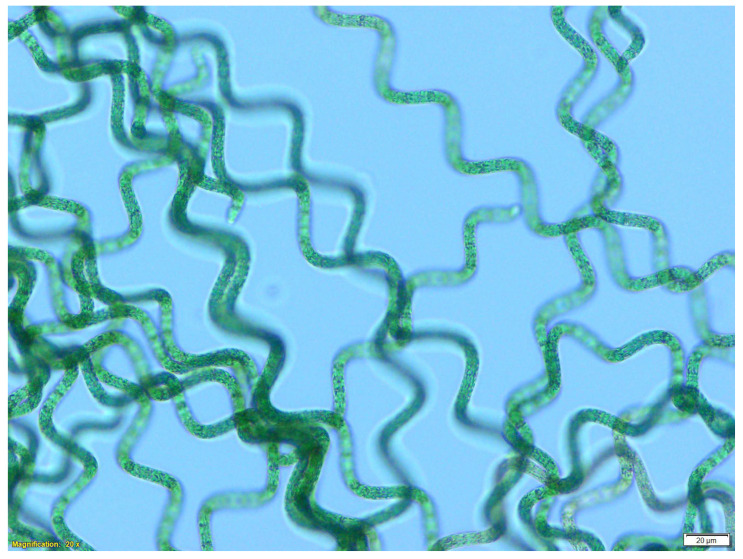
*Arthrospira platensis* ULC 445 from S samples observed without staining procedures under the optical microscope Olympus BX53 (magnification 20×).

*Spirulina* remained viable only in the S samples, that is, the *Spirulina* spheres stored in the dark at 4° not in contact with the yogurt. In contrast, the Y+S (in contact with the yogurt) were able to grow at the start of the trial, that is, spheres that were produced, added to the yogurt, and immediately taken for analysis. But the spheres did not survive in contact with yogurt as the analysis time progressed. Irrespective of time, Y+S samples at 0 days and all S samples showed significant differences (*p* < 0.05) compared to the other samples. The optical density (OD) results were matched with macroscopic and microscopic observation of the different *Spirulina* cultures up to 17 days. As time progressed, the inoculated *Spirulina* samples showed the presence of biomass of the typical green-blue color in contrast to the uninoculated Zarrouk medium that remained clear. Therefore, the OD values refer to the presence of viable cells. Figure 4 shows the microscopic images of *Spirulina* taken from S samples during the viability test. As can be observed, the cells have the typical spiral shape and blue-green color, which indicates the healthy state of the culture.

## 4. Discussion

The ever-growing interest both in functional food and food supplements led us to formulate a fresh *Spirulina* biomass embedded into alginate spheres. These spheres may potentially remain viable and be used either by consumers as a supplement for yogurt or other foods and drinks, or by companies to produce fortified foods/drinks.

This study involved two aspects: the *Spirulina* retained viability in S samples and the potential compound release in Y+S samples.

Fresh *Spirulina* alginate spheres were obtained by direct spherification technique. The alginate (1–4%) and the cross-linker concentrations are important factors affecting the structure of the spheres. The qualitative characteristics of the *Spirulina* spheres here produced are in agreement with Bennacef et al. [23], who reported that the higher the concentration of alginate the greater the hardness and thickness of the spheres while the greater the concentration of calcium the greater the fragility of the spheres. Although the sphere characteristics derived from qualitative observations, they were useful in choosing the ratio of sodium alginate-calcium lactate to use for the spherification process of the main trial.

The incorporation of *Spirulina* spheres was found to stimulate the growth of both *L. bulgaricus* and *S. thermophilus* throughout the storage time, corroborating previous findings on the prebiotic potential of *Spirulina*-derived compounds. The results regarding pH and LAB are consistent with those reported by other authors. Indeed, the reduction in pH in samples enriched with *Spirulina* was justified by the fact that the microalga promotes the development of LAB in yogurt and consequently acidification [24,25]. Our results regarding the stimulatory effect of *Spirulina* spheres on LAB are in agreement with several authors who highlighted that *Spirulina*, even in a non-spherical form, when added to fermented dairy products, favors the development of LAB [25,26,27,28,29]. Indeed, *Spirulina* can act as prebiotic stimulating the LAB due to minerals, vitamins, free amino acids, phenolic compounds, and exopolysaccharides [30,31,32,33]. In particular, oligosaccharides such as rhamnose, mannose, xylose, and galactose produced by *Spirulina* are reported as LAB booster [34,35,36]. Dried or fresh *Spirulina* at different concentrations positively affects LAB growth: Bchir [12] reports that the addition of 0.5% fresh *Spirulina* to yogurt gave the highest *S. thermophilus* loads after 15 d of storage as compared to yogurt with the addition of dried *Spirulina* and to unfortified yogurt. The addition of 1% of *Spirulina* powder to yogurt determined, after one week, higher *Lactobacillus acidophilus* loads (7.92 Log CFU/mL) compared to the control yogurt [37]. The addition of 0.8% of *Spirulina* powder to yogurt led to higher *L. bulgaricus* (7.0 Log CFU/mL) and *S. thermophilus* (8.5 Log CFU/mL) counts compared to the unfortified product [24]. Similar results for *S. thermophilus* are reported with the addition of 0.6% of *Spirulina* powder in fermented milks [38]. *Spirulina* powder (0.5–1%) added to yogurt had a positive effect on *L. delbrueckii* subsp. *bulgaricus* and *S. thermophilus*, whose concentration was higher than 10^6^ CFU/g compared to the control yogurt [31]. Yogurt enriched with *Spirulina* powder exhibited higher loads of probiotic *Lactobacillus acidophilus* and *Bifidobacterium animalis* compared to the unenriched yogurt [39]. Dried *Spirulina* has been reported as a suitable growth substrate for *Lactiplantibacillus plantarum* 8014 since after 72 h it reached a concentration about of 10^5^ Log CFU/mL in soybean drink with added *Spirulina* [40]. To the best of our knowledge, studies concerning the addition of encapsulated dried *Spirulina* have regarded the physicochemical, antioxidant, and sensory aspects of yogurts [7,20,41]. Recently, Yağmur [42] has reported the LAB stimulatory effect of microcapsules containing pomegranate and *Spirulina* extracts added to yogurts, while, as previously said, no papers have reported the encapsulation of fresh *Spirulina* and its use to fortify yogurt. To validate the hypothesis that the LAB booster effect observed in the present paper by the fresh *Spirulina* spheres is determined by the release into the yogurt of *Spirulina* bioactive compounds, future investigations on their chemical quantification are needed.

Alginate gel has a porous nature that allows for the diffusion of compounds in or out of the spheres. The higher the density of the alginate, the lower the permeability [43]. Another study has reported the role of the calcium chloride in substance diffusion; the higher its concentration, the lower the release [44]. Also, it was reported that release of *Spirulina* from spheres occurs by alginate matrix erosion [45]. Moreover, in a low-pH environment the alginate matrix undergoes shrinking with a decrease in pore size [43], which consequently affects the diffusion of compounds. The interaction of the above-reported mechanisms may explain the effect of *Spirulina* spheres on the LAB as a consequence of the release of *Spirulina* and its bioactive compounds into the yogurts; as stated above, their role needs to be verified in order to validate both this hypothesis and the production methodology.

The OD measurement is reported as a method to estimate microalgae biomass and used to monitor the growth of cells over time [46,47]. OD does not measure the percentage of living cells, but increasing OD values indicate that the culture is growing and viable. Therefore, the increasing OD values of the *Spirulina* spheres inoculated in fresh Zarrouk, together with the macroscopic observation of the cultures showing presence of biomass, proved the viability of the *Spirulina* embedded in the alginate spheres not in contact with yogurt. On the other hand, the observed loss of viability of *Spirulina* in Y+S samples is probably due to the excessively low pH conditions of the yogurt detected throughout the 15 days compared to the optimum range of 8.5–11 for *Spirulina* [48]. As reported above, a possible cause of the death of the sphere-embedded *Spirulina* in contact with yogurt is the diffusion of the acids inside the spheres, due to the permeability of the alginate matrix. Although contact with yogurt ultimately leads to the death of the *Spirulina* in the spheres, its bioactive molecules should remain unaltered since the biomass of *Spirulina* used was fresh and viable, and no processes able to cause compound degradation, such as high temperatures, were used. This conclusion needs to be further verified by molecular investigations. Consequently, the loss of viability of *Spirulina* within the yogurt matrix may not be detrimental, as the release of bioactive compounds could still confer functional benefits.

In order to protect the viability of *Spirulina*, potential future improvements could be to vary the parameters of spherification, such as sodium alginate and calcium lactate concentration and/or coating the alginate spheres using polycations, such as chitosan, or hydrophobic waxes that form an outer layer on the surface of the spheres and confer a barrier against the diffusion of the compounds in and out of the spheres [49,50,51,52,53].

An important step for novel food products is the sensory analysis that gives information on their taste, aroma, texture, appearance, and global acceptance by consumers [54]. Therefore, future sensory investigations are paramount to ascertain not only consumers’ acceptance of the novel yogurt enriched with fresh *Spirulina* spheres but also consumers’ propensity to use the novel *Spirulina* formulation to fortify any food preparation.

## 5. Conclusions

The novelty of this study focused on the maintenance of the viability of *Spirulina* embedded in alginate spheres, as well as their addition to yogurt. The continued viability of these spheres, when stored in the dark at 4 °C and not in contact with yogurt, demonstrated the technical feasibility of this new formulation of fresh *Spirulina*, which can be refrigerated and added to any food preparation.

While *Spirulina* viability was compromised in the yogurt matrix, this may still offer benefits due to the potential release of bioactive compounds. On the other hand, changing the spherification parameters could lead to the production of impermeable spheres that prevent the acidity of the yogurt from damaging the *Spirulina*. Further studies about the release and the maintenance of the compounds’ bioactivity are required to prove their potential functional benefit both for S and Y+S *Spirulina* spheres. Although, the visual appearance of the novel fortified yogurt with fresh *Spirulina* spheres suggests potential for improved consumer acceptability, this needs to be ascertained by panel test validation. Optimizing the spherification parameters, the controlled release of the compounds, and the sensory evaluation of the lab-scale novel products will be useful starting points for industrial scalability. Also necessary for large scale production is the adaptation of the *Spirulina* growth parameters, so as to process large volumes of *Spirulina* culture in reactors of progressively larger dimensions, and a scaling-up of the spherification system. Overall, this study demonstrates a promising strategy for the incorporation of fresh *Spirulina* into functional foods. Further research is warranted to optimize encapsulation parameters, confirm compound bioavailability, and assess sensory acceptability for potential industrial applications.

## Figures and Tables

**Table 1:** Acronyms of the different sample types.

Samples	Acronyms
Unenriched *Spirulina* yogurt	Y
*Spirulina* spheres	S
Yogurt enriched with *Spirulina* spheres	Y+S

**Table 2:** Diameter and qualitative characteristics of *Spirulina* spheres obtained by mixing two concentrations of sodium alginate and two concentrations of calcium lactate.

Spheres Types	Diameters (mm)	Qualitative Characteristics
SA 3%—CL 2.5%	8.8 ± 0.02 ^a^	completely solidified and hard
SA 3%—CL 1.5%	5.0 ± 0.01 ^b^	thin external film, fairly resistant
SA 2%—CL 2.5%	4.6 ± 0.02 ^c^	thin external film, fairly resistant
SA 2%—CL 1.5%	5.1 ± 0.02 ^b^	easily breakable

SA: sodium alginate; CL: calcium lactate. Values are mean and standard deviation (*n* = 20). Means with different lowercase superscripts are significantly different (*p* < 0.05).

**Table 3:** pH values of the yogurt (Y) and yogurt fortified with *Spirulina* spheres (Y+S) monitored across 15 days.

Samples	Days					
	0	2	6	8	10	15
Y	4.26 ± 0.00 ^a^	4.12 ± 0.01 ^bc^	4.10 ± 0.01 ^cd^	4.11 ± 0.00 ^cd^	4.11 ± 0.01 ^cd^	4.04 ± 0.00 ^e^
Y+S	4.26 ± 0.01 ^a^	4.14 ± 0.01 ^b^	4.11 ± 0.01 ^cd^	4.14 ± 0.00 ^b^	4.14 ± 0.01 ^b^	4.08 ± 0.01 ^d^

Values are mean ± standard deviation. Means with different superscript letters are significantly different (*p* < 0.05).

**Table 4:** Growth of *Spirulina* embedded in S and Y+S spheres by spectrophotometric analysis (OD_550nm_) cultured in Zarrouk medium at the start of the trials and at two days after each analysis time (2, 6, 8, 10, and 15 days).

Samples	Days						
	0	2	4	8	10	12	17
S	0.254 ± 0.016 ^a^	0.256 ± 0.021 ^a^	0.250 ± 0.007 ^a^	0.252 ± 0.011 ^a^	0.246 ± 0.012 ^a^	0.255 ± 0.009 ^a^	0.2515 ± 0.005 ^a^
Y+S	0.251 ± 0.003 ^a^	0.000 ± 0.000 ^b^	0.000 ± 0.000 ^b^	0.000 ± 0.000 ^b^	0.000 ± 0.000 ^b^	0.000 ± 0.000 ^b^	0.000 ± 0.000 ^b^
Zarrouk medium	0.000 ± 0.000 ^b^	0.000 ± 0.000 ^b^	0.000 ± 0.000 ^b^	0.000 ± 0.000 ^b^	0.000 ± 0.000 ^b^	0.000 ± 0.000 ^b^	0.000 ± 0.000 ^b^

Values are mean ± standard deviation. Means with different superscript letters are significantly different (*p* < 0.05).

## Data Availability

The original contributions presented in this study are included in the article. Further inquiries can be directed to the corresponding author.
